# Causes of Fever in a Cohort of Nepali Children and the Potential Impact of Molecular Testing During a Dengue Fever Outbreak

**DOI:** 10.1097/INF.0000000000005167

**Published:** 2026-01-30

**Authors:** Peter J. O’Reilly, Madhav C. Gautam, Bhishma Pokhrel, Sonu Shrestha, Meeru Gurung, Sanjeev M. Bijukchhe, Elizabeth O’Mahony, Catherine Davis, Andrew Taylor, Sarah Kelly, Ruby Basi, Anushiya Kattel, Kushal Gautam, Shriya Bista, Roshan Jha, Ram Khadka, Saugat Bhandari, Puja Amatya, Ganesh Shah, Ira Shrestha, Michael Carter, Shreekrishna Maharjan, Colin Fink, Michael Levin, Aubrey J. Cunnington, Andrew J. Pollard, Shrijana Shrestha, on behalf of the DIAMONDS Consortium, Giselle D’Souza

**Affiliations:** From the * Oxford Vaccine Group, Department of Paediatrics, University of Oxford, Oxford, UK; † Patan Academy of Health Sciences, Lalitpur, Nepal; ‡ Micropathology Ltd., University of Warwick Science Park, Coventry, UK; § NIHR Oxford Biomedical Research Centre, Oxford, UK; ¶ Centre for Human Genetics, University of Oxford, Oxford, UK; ∥ Department of Infectious Disease, Centre for Paediatrics and Child Health, Imperial College, London, UK.

**Keywords:** polymerase chain reaction, molecular, Nepal, dengue, diagnostics

## Abstract

**Background:**

Identifying the cause of infection is important for clinical management and public health decisions, including vaccination strategies. In low-resource settings, causes of fever are often not identified. In this study, molecular testing panels were used to identify the causes of pediatric fever in the Kathmandu Valley, Nepal. A dengue fever outbreak facilitated the investigation of dengue diagnostics.

**Methods:**

Children under 14 years of age were recruited to this prospective cohort study at Patan Hospital, Nepal. Clinical data and routine diagnostics were used to classify cases, including nonstructural protein 1 (NS1) antigen testing for dengue. Additional molecular diagnostics were performed on blood (12 viral, 26 bacterial and 6 fungal targets) and respiratory samples (17 viral and 3 bacterial targets).

**Results:**

From September 1, 2021, to April 19, 2023, 565 children were enrolled, median age 3 (interquartile-range 1–7) years. Pathogens identified included dengue virus (n = 101), respiratory syncytial virus (n = 30), influenza (n = 25), typhoidal *Salmonella* spp. (n = 7) and *Neisseria meningitidis* (n = 2). During the dengue outbreak, dengue polymerase chain reaction (PCR) and NS1 positivity rates were both high early in dengue disease, but if >3 days of symptoms, PCR positivity rates declined (10.3%) while NS1 positivity remained high into the second week of illness (80%).

**Conclusions:**

This prospective cohort study is the most comprehensive effort to date to describe the causes of pediatric fever in the Kathmandu Valley, Nepal. The United States Centers for Disease Control and Prevention recommends dengue PCR or NS1 antigen testing during the first 7 days of dengue fever. Our data indicate that PCR positivity declines after 3 days of symptoms, resulting in missed cases when relying solely on PCR.

In low-resource settings, causes of fever are often not identified, with a lack of basic microbiologic testing in many settings.^1^ Identifying the causative pathogen is important for individual management decisions, reducing antimicrobial use and providing public health information.^2^

In Nepal, infections are the most common causes of pediatric hospitalizations; however, there are few data regarding which pathogens cause febrile illnesses. During seasonal outbreaks, dengue fever is an important cause of fever in Nepali children.^3^ International guidance recommends that polymerase chain reaction (PCR) or nonstructural protein 1 (NS1) antigen testing can be used to diagnose dengue.^4,5^ Antigen-based rapid diagnostic testing for NS1 enables bedside testing for dengue infection but may have low specificity.^6^ Molecular testing is considered the most sensitive and specific test available for dengue fever, but is not part of routine testing in many settings, including Patan Hospital, where this study was set.^7^

The study hypothesis is that molecular testing would improve diagnostic yield in pediatric fever cases. The primary objective of this study was to describe the causes of pediatric fever in the Kathmandu Valley using additional molecular testing panels. A dengue fever outbreak during enrollment provided the opportunity to investigate dengue diagnostics.

## MATERIALS AND METHODS

### Setting

A prospective study was undertaken at Patan Hospital, a 640-bed teaching hospital, which serves a large urban population in the Kathmandu Valley, Nepal.

The study is part of a larger multisite consortium project with broad diagnostic research aims (see diamonds2020.eu https://links.lww.com/INF/G545). Ethical approval was obtained from the Ethical Review Board of the Nepal Health Research Council, the Institutional Review Committee of Patan Academy of Health Sciences and from the University of Oxford’s Tropical Research Ethics Committee.

### Study Procedures

Any child, up to 14 years of age, presenting to Patan Hospital with signs of infection was eligible for enrollment. Preterm neonates, with a corrected gestational age of less than 37 weeks, were excluded. Informed consent was obtained from the parents/guardians of all participants.

A research blood sample was taken as soon as feasible after presentation to the hospital. Blood collected in an ethylenediaminetetraacetic acid tube was used for molecular testing. A nasopharyngeal swab sample was obtained and placed in a cryovial with transport medium containing skim milk, tryptone, glucose and glycerol. See Text, Supplemental Digital Content 2, https://links.lww.com/INF/G545.

**FIGURE 1.**
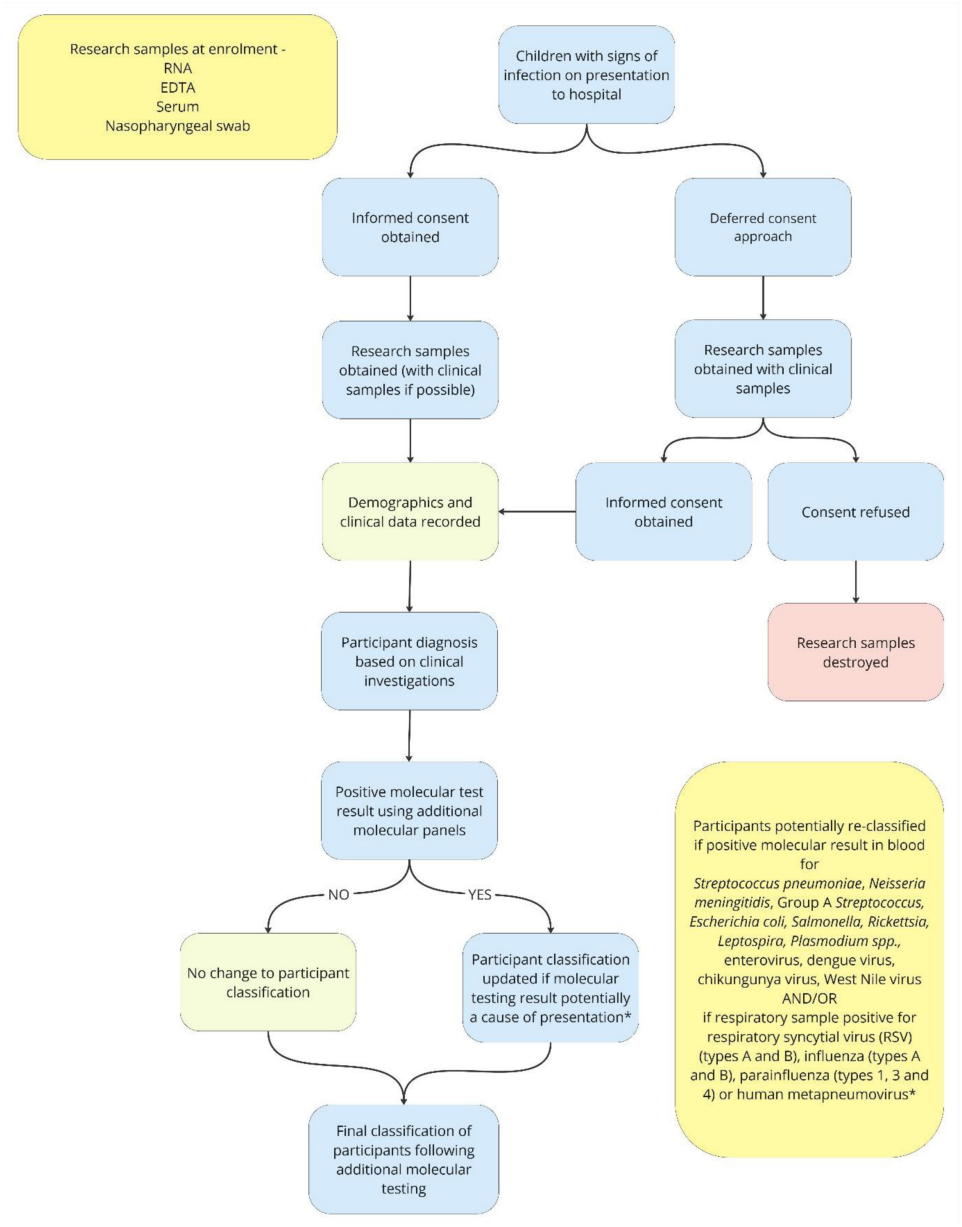
Flowchart of study procedures and use of additional molecular testing panels to reclassify participants.

Demographic information and details of the clinical presentation were collected. Medical charts were reviewed. Data were collected on investigations, treatments provided and outcomes. A flow chart showing the study procedures is provided in Figure 1.

### Laboratory Procedures

See Text, Supplemental Digital Content 2 https://links.lww.com/INF/G545, for details of laboratory procedures. In brief, an aliquot of whole blood and skim milk, tryptone, glucose and glycerol media from each nasopharyngeal sample were sent to Micropathology Ltd., University of Warwick Science Park, United Kingdom, for molecular testing.

Nucleic acid from the nasopharyngeal samples was analyzed using the NxTAG Respiratory Pathogen Panel +SARS-CoV-2 (Luminex Corporation). This panel detects 17 different viral targets and 3 bacterial targets. Extracted nucleic acid from blood samples was analyzed using a molecular diagnostic panel developed and validated at Micropathology Ltd. This panel detects 9 different viruses (9 viral targets), 16 bacteria (23 targets) and 2 fungal species (6 fungal targets).^8^

Plasma aliquots (100 μL) were tested using the Siemens Tropical Fever Core Panel at Patan Hospital. Samples that tested positive for dengue on PCR were tested using the Siemens Dengue Differentiation Panel, with targets for the four dengue serotypes. See Supplemental Digital Content 3, https://links.lww.com/INF/G545, for the pathogen target list.

### Additional Molecular Testing

Only a subset of the molecular targets has been associated with clinical disease in case–control studies, and these targets were used to identify additional causes of infection.^9^ See Supplemental Digital Content 4, https://links.lww.com/INF/G545, for the evidence for using specific molecular targets and statistical methods.

Positive molecular results for bacterial targets *Neisseria meningitidis*, *Streptococcus pneumoniae*, *Escherichia coli*, Group A *Streptococcus, Rickettsial* spp., *Leptospira* spp. or *Salmonella* spp. were taken as possibly clinically significant. The viral targets used as possibly clinically significant were dengue virus and enterovirus in blood, and respiratory syncytial virus (types A and B), influenza (types A and B), parainfluenza (types 1, 3 and 4), and human metapneumovirus in nasopharyngeal samples.

When a molecular test was positive for a potential pathogen, the clinical case was reviewed to ensure the molecular result was potentially a cause of the presentation. When the molecular result did not fit with the clinical presentation, these results were ignored.

## RESULTS

### Epidemiologic Data

Between September 1, 2021, and April 19, 2023, 574 participants were enrolled. The median age was 3 years (interquartile range 1.05–6.96 years), and 35.2% of the cohort were female.

### Molecular Diagnostics

Combining routine diagnostics with additional molecular testing panels, 44% (220/498) of participants had a potential causal pathogen identified.

The most common pathogens identified were viruses, including dengue virus (n = 101), respiratory syncytial virus (n = 30), influenza (n = 25), severe acute respiratory syndrome coronavirus 2 (n = 17), human metapneumovirus (n = 14) and hepatitis A (n = 4). Important bacterial causes identified included *E. coli*-positive urine cultures (n = 20), as well as blood, pleural fluid and/or cerebrospinal fluid samples positive for *S. pneumoniae* (n = 4), *Salmonella* Paratyphi A (n = 4), *Salmonella* Typhi (n = 3) and *Mycobacterium tuberculosis* (n = 2) (see Supplemental Digital Content 5, https://links.lww.com/INF/G545).

### Dengue Outbreak

During the study period, there was a dengue fever outbreak in the Kathmandu Valley. Between the diagnosis of the first dengue case in the study, August 4, 2022, and the final dengue case diagnosed, November 19, 2022, there were 176 participants enrolled; 139 of these participants (79%) had at least 1 dengue test performed, 101 of these participants tested positive for dengue virus using PCR, NS1 antigen test, dengue-specific immunoglobulin (Ig)M or IgG. One participant had dengue testing performed outside of Patan Hospital and was excluded from the analyses.

### Dengue Molecular Testing

As part of routine care, NS1 testing results were available for 56.8% (100/176) of participants during the outbreak period, and 85% (85/100) of NS1 tests were positive. As part of the additional molecular testing, PCR was carried out on 48.3% (85/176) of samples, and 31.8% (27/85) of samples were PCR-positive.

**FIGURE 2.**
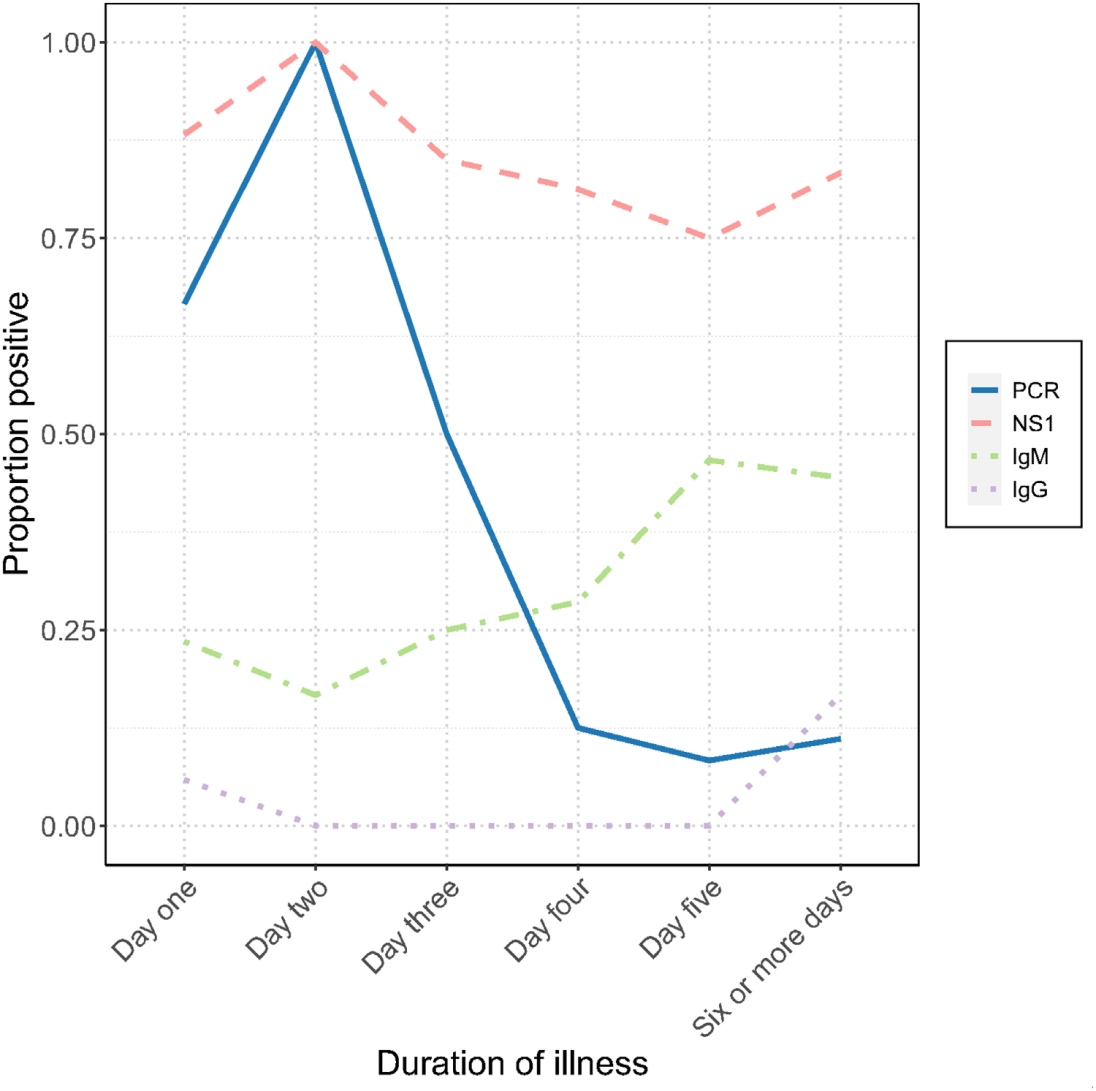
Proportion of positive test results for dengue virus among the participants with a diagnosis of dengue. The proportion of positive tests for dengue polymerase chain reaction (PCR), nonstructural protein 1 (NS1) antigen, dengue-specific immunoglobulin M (IgM) and dengue-specific immunoglobulin G (IgG) are presented. The X-axis represents the number of days of illness when the sample was taken for testing.

Of the 72 participants that had results for PCR and NS1 testing, 1 case was PCR-positive and NS1-negative, whereas 32 participants were NS1-positive and PCR-negative. The duration of dengue fever symptoms affected the positivity rates (Fig. 2). In the first 2 days of illness, NS1 (93%, 28/30) and PCR (78%, 18/23) had high positivity rates; however, from day 3 onwards the proportion of PCR-positive samples decreased.

In participants presenting with 4 or more days of symptoms, the proportion of PCR-positive results was 10.4% (3/29). The proportion of NS1-positive participants was 80% (40/50) in the same participants with 4 or more days of symptoms.

### Dengue Serotypes

Dengue PCR-positive samples were tested for dengue serotypes. Serotype 1 was most often detected, 66.7% (12/18), followed by serotype 3, 16.7%, (3/18), and serotype 2, 11.1% (2/18). Serotype 4 was not detected.

## DISCUSSION

This study is the most comprehensive effort to date to describe the causes of pediatric fever in the Kathmandu Valley. Many dengue cases were identified, which allowed for an investigation of dengue diagnostics.

Identifying the causes of fever in a population is important. Clinicians can use these data to help when making management decisions in individual cases. For public health teams, knowing the causes of pediatric infections is important when making vaccine program decisions. Even with additional molecular testing, a causative pathogen was not identified in over half of cases. This highlights the need for ongoing research to improve diagnostics in pediatric infection.

Study limitations include potential selection bias, as patients with severe disease due to rare pathogens may not have been included in the study, as obtaining research samples can be difficult when children are severely unwell. Over 500 participants were enrolled in the study; however, rare causes of fever may have been missed due to sample-size constraints. Although the Tropical Fever Panel testing was carried out locally at Patan Hospital, other molecular testing was carried out in the United Kingdom; molecular testing panels that can be performed locally would be more useful if these tests are to be used for diagnostic purposes.

Many participants with dengue fever were enrolled. The Kathmandu Valley has historically been spared from dengue virus outbreaks, but changes in climate are allowing for the spread of arboviruses to new areas.^3^ Dengue serotypes 1, 2 and 3 were identified during the outbreak, with serotype 1 being the most common. Identifying circulating dengue serotypes is important for vaccine implementation and monitoring. Changes in circulating serotypes are important; the risk of severe dengue disease increases when an individual has a second infection with a different serotype.^10^

Regarding dengue diagnostics, NS1 testing was the most common method of confirming infection. PCR is considered the most sensitive and specific test, especially early in dengue infection, but PCR is not routinely available at Patan Hospital.^7^

In a study of admitted dengue cases in Pakistan, Iqbal et al. reported that 28.5% of NS1-positive results were false positives when taking PCR as the gold standard.^6^ Rather than the NS1 results being false positives, the duration of symptoms at the time of testing likely contributed to differences between NS1 and PCR results.

Knowing when to expect different dengue tests to be positive is important in making an accurate diagnosis. The United States Centers for Disease Control and Prevention recommends using PCR or NS1 testing, from 0 to 7 days of symptoms.^4^ Our study shows high PCR positivity early in infection, but PCR is likely to be negative by day 4 of disease. Days 4–6 of symptoms are the critical phase for dengue infection, when severe dengue shock may present.

The European Centre for Disease Prevention and Control’s Factsheet for Health Professionals suggests that the NS1 antigen can be detected until the fourth day of illness.^5^ Our data show NS1 antigen remains positive into the second week of dengue illness. Persisting NS1 positivity could create diagnostic problems in the setting of a child presenting with a serious bacterial illness following a recent dengue infection. Our data suggest that there are potential issues with relying on PCR or NS1 testing alone for the diagnosis of acute dengue.

This study describes the causes of pediatric fever in the Kathmandu Valley. These data add useful information for clinicians involved in clinical management of pediatric infections; public health officials can use these data to aid in decisions on the introduction of new vaccines, for example, dengue fever vaccines. We identified a small number of molecular tests, which could improve the diagnosis of pediatric fever in Kathmandu. Future studies, using these potentially useful molecular tests in real time to influence diagnosis, should be carried out to confirm the utility of these molecular tests.

Our data highlight potential benefits and limitations of PCR and NS1 antigen testing in dengue fever. These data can be used by policymakers to provide more accurate information to clinicians on how to interpret dengue fever diagnostics.

## Supplementary Material



## References

[1] Bottieau E Van Duffel L Safi S E. Etiological spectrum of persistent fever in the tropics and predictors of ubiquitous infections: a prospective four-country study with pooled analysis. BMC Med. 2022;20:144. 35491421 10.1186/s12916-022-02347-8 PMC9059373

[2] Suneja M Beekmann SE Dhaliwal G. Diagnostic delays in infectious diseases. Diagnosis (Berl). 2022;9:332–339. 35073468 10.1515/dx-2021-0092 PMC9424060

[3] Amatya B Schwartz E Biber A. Dengue serotype characterization during the 2022 dengue epidemic in Kathmandu, Nepal. J Travel Med. 2023;30:taad034. 36971480 10.1093/jtm/taad034

[4] United States Centers for Disease Control and Prevention. Clinical Testing Guidance for Dengue; 2024. Available at: https://www.cdc.gov/dengue/hcp/diagnosis-testing/index.html. Accessed 9th Jan 2025.

[5] European Centre for Disease Prevention and Control. Factsheet for Health Professionals About Dengue; 2023. Available at: https://www.ecdc.europa.eu/en/dengue-fever/facts. Accessed 9th Jan 2025.

[6] Iqbal G Javed H Raza FA. Diagnosis of acute dengue virus infection using enzyme-linked immunosorbent assay and real-time PCR. Can J Infect Dis Med Microbiol. 2023;2023:3995366. 37261378 10.1155/2023/3995366 PMC10228213

[7] World Health Organisation. WHO Guidelines Approved by the Guidelines Review Committee. Dengue: Guidelines for Diagnosis, Treatment, Prevention and Control: New Edition. World Health Organization; 2009.

[8] Shah P Voice M Calvo-Bado L; PERFORM consortium. Relationship between molecular pathogen detection and clinical disease in febrile children across Europe: a multicentre, prospective observational study. Lancet Reg Health Eur. 2023;32:100682. 37554664 10.1016/j.lanepe.2023.100682 PMC10405323

[9] O’Brien KL Baggett HC Brooks WA. Causes of severe pneumonia requiring hospital admission in children without HIV infection from Africa and Asia: the PERCH multi-country case-control study. Lancet. 2019;394:757–779. 31257127 10.1016/S0140-6736(19)30721-4 PMC6727070

[10] Bhatt P Sabeena SP Varma M. Current understanding of the pathogenesis of dengue virus infection. Curr Microbiol. 2021;78:17–32. 33231723 10.1007/s00284-020-02284-w PMC7815537

