# Supplementary material for: Causes of Fever in a Cohort of Nepali Children and the Potential Impact of Molecular Testing During a Dengue Fever Outbreak
Source: Pediatr Infect Dis J. 2026 Jan 30;45(7):e224–8. doi: 10.1097/INF.0000000000005167 (PMC13232699; doi:10.1097/INF.0000000000005167)
Supplement: Supplementary file 1 [file inf-45-e224-s001.pdf]

SUPPLEMENTAL DIGITAL CONTENT 1. DIAMONDS Consortium Members List

PARTNER: Imperial College (Coordinating Centre) (UK)

Chief investigator/DIAMONDS coordinator: Michael Levin[1]

Principal and co-investigators (alphabetical order):[1] Aubrey Cunnington; Jethro Herberg; Myrsini Kaforou; Victoria J. Wright

Section of Paediatric Infectious Diseases Research Group (alphabetical order):[1] Evangelos Bellos; Claire Broderick; Samuel Channon-Wells; Samantha Cooray; Tisham De (database work package lead); Giselle D'Souza; Amedine Duret; Ankita Duseja; Leire Estramiana Elorrieta; Diego Estrada-Rivadeneyra; Rachel Galassini (Clinical Trial Manager); Dominic Habgood-Coote; Shea Hamilton (Proteomics); Heather Jackson; James Kavanagh; Ilana Keren; Mahdi Moradi Marjaneh; Stephanie Menikou; Samuel Nichols; Ruud Nijman; Harsita Patel; Ivana Pennisi; Oliver Powell; Ruth Reid; Priyen Shah; Ortensia Vito; Elizabeth Whittaker; Clare Wilson; Rebecca Womersley

Recruitment team at Imperial College Healthcare NHS Trust, London (alphabetical order):[2] Amina Abdulla; Sarah Darnell; Sobia Mustafa

Engineering Team: Pantelis Georgiou[3] (engineering lead); Jesus Rodriguez-Manzano[4]; Nicolas Moser[3]; Ivana Pennisi[1]

[1]Section of Paediatric Infectious Disease, Imperial College London, Norfolk Place, London W2 1PG, UK

[2]Children's Clinical Research Unit, St Mary's Hospital, Praed Street, London W2 1NY, UK

[3] Imperial College London, Department of Electrical and Electronic Engineering, South Kensington Campus, London, SW7 2AZ, UK

[4] Imperial College London, Department of Infectious Disease, Section of Adult Infectious Disease, Hammersmith Campus, London, W12 0NN, UK

UK Non-Consortium Clinical Recruiting Sites

Evelina London Children's Hospital, Guy's and St Thomas' NHS Foundation Trust; King's College London [combined]

Michael Carter[1,2] and Paul Wellman[1]; (principal investigator); Shane Tibby[1,2] (co-investigator)

Recruitment team (alphabetical order): Jonathan Cohen[1]; Francesca Davis[1;] Julia Kenny[1]; Marie White[1]

Laboratory team (alphabetical order): Matthew Fish[3]; Aislinn Jennings[4]; Manu Shankar-Hari[3,4]

[1] Evelina London Children's Hospital, Guy's and St Thomas' NHS Foundation Trust, London, UK

[2] Department of Women and Children's Health, School of Life Course Sciences, King's College London, UK

[3] Department of Infectious Diseases, School of Immunology and Microbial Sciences, King's College London, London, UK

[4] Department of Intensive Care Medicine, Guy's and St Thomas' NHS Foundation Trust, London, UK

University Hospitals Sussex

Katy Fidler[1] (principal investigator); Dan Agranoff[2] (co-investigator)

Recruitment team; Vivien Richmond[1,3], Mathhew Seal[2]

[1] Royal Alexandra Children's Hospital, University Hospitals Sussex, Brighton, UK

[2] Dept of Infectious Diseases, University Hospitals Sussex, Brighton, UK

[3] Research Nurse team, University Hospitals Sussex, Brighton, UK

University Hospital Southampton NHS Foundation Trust

Saul Faust[1] (principal investigator); Dan Owen[1] (co-investigator);

Recruitment team; Ruth Ensom[2]; Sarah McKay[2]; Diana Mondo[3], Mariya Shaji[3]; Rachel Schranz[3] *(alphabetical order)*

[1] NIHR Southampton Clinical Research Facility, University Hospital Southampton NHS Foundation Trust and University of Southampton, UK

[2] NIHR Southampton Clinical Research Facility, University Hospital Southampton NHS Foundation Trust, UK

[3] Department of R&D, University Hospital Southampton NHS Foundation Trust, UK

Barts Health NHS Trust

Prita Rughani[1, 2, 3] (principal investigator 2020-2021); Amutha Anpananthar[1, 2, 3] (principal investigator 2021-to date); Susan Liebeschuetz[2] (co-investigator), Anna Riddell[1] (co-investigator)

Recruitment team; Divya Divakaran[3,] Louise Han[3,], Nosheen Khalid[1, 3,]Ivone Lancoma-Malcolm, Jessica Schofield[3,] Teresa Simagan[3] *(alphabetical order)*

[1] Royal London Hospital, Whitechapel Rd, London E1 1FR, UK

[2] Newham University Hospital, Glen Rd, London E13 8SL, UK

[3] Whipps Cross University Hospital, *Whipps Cross* Road, London, E11 1NR, UK

Great Ormond Street Hospital for Children NHS Foundation Trust

Mark Peters[1,2] (principal investigator); Alasdair Bamford[1,2] (co-investigator)

Recruitment team; Lauran O'Neill[1]

[1] Great Ormond Street Hospital, London, WC1N 3JH, UK

[2] UCL Great Ormond St Institute of Child Health, WC1N 1EH, UK

Cambridge University Hospitals NHS Foundation Trust

Nazima Pathan[1,2] (principal investigator)

Recruitment team; Esther Daubney[1], Deborah White[1] (alphabetical order)

[1]Addenbrooke's Hospital, Hills Road, Cambridge CB2 0QQ, UK

[2]Department of Paediatrics, University of Cambridge, Cambridge CB2 0QQ, UK

University College London Hospitals NHS Foundation Trust

Melissa Heightman[1] (principal investigator); Sarah Eisen[1] (co-investigator)

Recruitment team; Terry Segal[1], Lucy Wellings[1] *(alphabetical order)*

[1] University College London Hospital, Euston Road, London NW1 2BU, UK

St George's University Hospitals NHS Foundation Trust

Simon B Drysdale[1] (principal investigator)

Recruitment team; Nicole Branch[1], Lisa Hamzah[1], Heather Jarman[1] (alphabetical order)

[1] St George's Hospital, Blackshaw Road, London SW17 0QT, UK

Lewisham and Greenwich NHS Trust

Maggie Nyirenda[1, 2,](principal investigator)

Recruitment team Lisa Capozzi[1], Emma Gardiner[1] *(alphabetical order)*

[1]University Hospital Lewisham, London SE13 6LH, UK

[2] Queen Elizabeth Hospital Greenwich, London SE18 4QH, UK

Liverpool University Hospitals NHS Foundation Trust

Robert Moots[1] (principal investigator); Magda Nasher[2] (principal investigator)

Recruitment team; Anita Hanson[2]; Michelle Linforth[1]

[1] Aintree University Hospital, Lower Lane, Liverpool L9 7AL, UK

[2] Royal Liverpool Hospital, Prescot St, Liverpool L7 8XP, UK

Leeds Teaching Hospitals NHS Trust

Sean O'Riordan[1] (principal investigator)

Recruitment team; Donna Ellis[1]

[1]Leeds Children's Hospital, Leeds LS1 3EX, UK

King's College Hospital NHS Foundation Trust

Akash Deep[1] (principal investigator)

Recruitment team; Ivan Caro[1]

[1] Kings College Hospital, Denmark Hill, London SE5 9RS, UK

Sheffield Children's NHS Foundation Trust

Fiona Shackley [1] (principal investigator);

Recruitment team; Arianna Bellini,[1] Stuart Gormley[1] *(alphabetical order)*

[1]Sheffield Children's Hospital, Broomhall, Sheffield S10 2TH, UK

University Hospitals of Leicester NHS Foundation Trust

Samira Neshat[1] (principal investigator)

[1]Leicester General Hospital, Leicester LE1 5WW, UK

Birmingham Women's and Children's Hospital NHS Foundation Trust

Barnaby J Scholefield[1] (principal investigator)

Recruitment team; Ceri Robbins[1], Helen Winmill[1] (alphabetical order)

[1] Birmingham Children's Hospital, Steelhouse Lane, Birmingham B4 6NH, UK

University of Oxford Partner

Children's Hospital, John Radcliffe Hospital, Oxford

Principal Investigator: Stéphane C. Paulus[1,2,3]

Co-Principal Investigator: Andrew J. Pollard[1,2,3,4]

Co-investigators: Mark Anthony[1] (neonates)

Recruitment team: Sarah Hopton[1], Danielle Miller[1], Zoe Oliver[1], Sally Beer[1], Bryony Ward[1]

[1]John Radcliffe Hospital, Oxford University Hospitals NHS Foundation Trust, Oxford, UK

[2]Department of Paediatrics, University of Oxford, UK

[3]Oxford Vaccine Group, University of Oxford, UK

[4]NIHR Oxford Biomedical Research Centre, Oxford, UK

University of Oxford, Nepal Site

Principal Investigator: Shrijana Shrestha[1]

Co-Principal Investigator: Andrew J Pollard[2,3]

Nepal Research Team: Meeru Gurung[1], Puja Amatya[1], Bhishma Pokhrel[1], Sanjeev Man Bijukchhe[1], Madhav Chandra Gautam [1]

Oxford Research Team: Sarah Kelly[2], Peter O'Reilly[2], Sonu Shrestha [2]

[1]Paediatric Research Unit, Patan Academy of Health Sciences, Kathmandu, Nepal.

[2]Oxford Vaccine Group, Department of Paediatrics, University of Oxford, Oxford, United Kingdom.

[3]NIHR Oxford Biomedical Research Centre, Oxford, United Kingdom.

SERGAS Partner (Spain)

Principal Investigators: Federico Martinón-Torres[1], Antonio Salas[1,2]

GENVIP RESEARCH GROUP (in alphabetical order): Fernando Álvez González[1], Sonia Ares Gómez[1], Xabier Bello[1,2], Mirian Ben García[1], Fernando Caamaño Viña[1], Sandra Carnota[1], María José Curras-Tuala[1,2], Ana Dacosta Urbieta[1], Carlos Durán Suárez[1], Isabel Ferreiros Vidal[1], Luisa García Vicente[1], Alberto Gómez-Carballa[1,2], Jose Gómez Rial[1], Pilar Leboráns Iglesias[1], Narmeen Mallah[1], Federico Martinón-Torres[1], Nazareth Martinón-Torres[1], José María Martinón Sánchez[1], Belén Mosquera Pérez[1], Jacobo Pardo-Seco[1,2], Sara Pischedda[1,2], Sara Rey Vázquez[1], Irene Rivero Calle[1], Carmen Rodríguez-Tenreiro[1], Lorenzo Redondo-Collazo[1], Antonio Salas[1,2], Sonia Serén Fernández[1], Marisol Vilas Iglesias[1].

[1] Translational Pediatrics and Infectious Diseases, Pediatrics Department, Hospital Clínico Universitario de Santiago, Santiago de Compostela, Spain, and GENVIP Research Group (www.genvip.org), Instituto de Investigación Sanitaria de Santiago, Universidad de Santiago de Compostela, Galicia, Spain.

[2] Unidade de Xenética, Departamento de Anatomía Patolóxica e Ciencias Forenses, Instituto de Ciencias Forenses, Facultade de Medicina, Universidade de Santiago de Compostela, and

GenPop Research Group, Instituto de Investigaciones Sanitarias (IDIS), Hospital Clínico Universitario de Santiago, Galicia, Spain

[3] Fundación Pública Galega de Medicina Xenómica, Servizo Galego de Saúde (SERGAS), Instituto de Investigaciones Sanitarias (IDIS), and Grupo de Medicina Xenómica, Centro de Investigación Biomédica en Red de Enfermedades Raras (CIBERER), Universidade de Santiago de Compostela (USC), Santiago de Compostela, Spain

Liverpool Partner

Principal Investigators: Enitan D Carrol[1,2,]

Research Group (in alphabetical order): Elizabeth Cocklin[1], Rebecca Beckley[1,2,3], Abbey Bracken[1], Ceri Evans[1,2], Aakash Khanijau[1], Rebecca Lenihan[1], Nadia Lewis-Burke[1], Karen Newall[3], Sam Romaine[1], Jennifer Whitbread [3]

[1] Department of Clinical Infection, Microbiology and Immunology, University of Liverpool Institute of Infection, Veterinary and Ecological Sciences , Liverpool, England

[2] Alder Hey Children's Hospital, Department of Infectious Diseases, Eaton Road, Liverpool, L12 2AP

[3]Alder Hey Children's Hospital, Clinical Research Business Unit, Eaton Road, Liverpool, L12 2AP

National and Kapodistrian University of Athens (Greece)

Principal Investigator: Maria Tsolia[1]

Co-Investigator: Irini Eleftheriou[1]

PID Unit: Nikos Spyridis[1], Maria Tambouratzi[1]

Pediatric Rheumatology Unit: Despoina Maritsi[1]

Lab: Antonios Marmarinos[1], Marietta Xagorari[1]

Recruitment teams: Adult COVID19- Infectious Diseases: Lourida Panagiota, Pefanis Aggelos[2]

Adult COVID19: Akinosoglou Karolina, Gogos Charalambos, Maragos Markos[3]

Adult Inflammatory Diseases-Oncology: Voulgarelis Michalis , Stergiou Ioanna[4]

[1]2nd Department of Pediatrics, National and Kapodistrian University of Athens (NKUA), Children's Hospital "P, and A. Kyriakou", Athens, Greece

[2]1st Department of Infectious Diseases, General Hospital "Sotiria"

[3]Pathology Department, University of Patras, General Hospital "Panagia i Voithia"

[4]Pathophysiology Department, Medical Faculty, National and Kapodistrian University of Athens (NKUA), General Hospital "Laiko"

Newcastle upon Tyne Hospitals NHS Foundation Trust and Newcastle University (UK) combined

Principal Investigator: Marieke Emonts [1,2,3] (all activities)

Co-investigators Emma Lim[2,3,6] (all activities), John Isaacs[1] (adult inflammatory)

Recruitment team (alphabetical), data managers, and GNCH Research unit: Kathryn Bell[4], Stephen Crulley[4], Daniel Fabian[4], Evelyn Thomson[4], Diane Wallia[4], Caroline Miller[4] , Ashley Bell[4]

PhD Students/medical staff DIAMONDS Fabian J.S. van der Velden[1,2] (all activities), Geoff Shenton[7] (oncology), Ashley Price[8,9] (Adult COVID)

Students Owen Treloar [1,2] (quality control, data management and analysis), Daisy Thomas[1,2] (recruitment)

Author Affiliations:

[1] Translational and Clinical Research Institute, Newcastle University, Newcastle upon Tyne UK

[2]Great North Children's Hospital, Paediatric Immunology, Infectious Diseases & Allergy, Newcastle upon Tyne Hospitals NHS Foundation Trust, Newcastle upon Tyne, United Kingdom.

[3]NIHR Newcastle Biomedical Research Centre based at Newcastle upon Tyne Hospitals NHS Trust and Newcastle University, Westgate Rd, Newcastle upon Tyne NE4 5PL, United Kingdom

[4]Great North Children's Hospital, Research Unit, Newcastle upon Tyne Hospitals NHS Foundation Trust, Newcastle upon Tyne, United Kingdom.

[6]Population Health Sciences Institute, Newcastle University, Newcastle upon Tyne, UK

[7]Great North Children's Hospital, Paediatric Oncology, Newcastle upon Tyne Hospitals NHS Foundation Trust, Newcastle upon Tyne, United Kingdom.

[8]Department of Infection & Tropical Medicine, Newcastle upon Tyne Hospitals NHS Foundation Trust, Newcastle upon Tyne, United Kingdom

[9]NIHR Newcastle In Vitro Diagnostics Co-operative (Newcastle MIC), Newcastle upon Tyne, United Kingdom.

Servicio Madrileño de Salud (SERMAS) - Fundación Biomédica del Hospital Universitario 12 de Octubre (FIB-H12O) (Spain)

Principal Investigators: Pablo Rojo[1,3], Cristina Epalza [1,2]

SERMAS/FIB-H120 team: Serena Villaverde[1], Sonia Márquez[2], Manuel Gijón [1,2], Romina Varchetta [2], Fátima Machín[2], Laura Cabello[2], Irene Hernández[2], Lourdes Gutiérrez[2], Ángela Manzanares [1,2]

Author Affiliations:

[1] Servicio Madrileño de Salud (SERMAS),Pediatric Infectious Diseases Unit, Department of Pediatrics, Hospital Universitario 12 de Octubre, Madrid, Spain

[2]Fundación Biomédica del Hospital Universitario 12 de Octubre (FIB-H12O), Unidad Pediátrica de Investigación y Ensayos Clínicos (UPIC), Hospital Universitario 12 de Octubre, Instituto de Investigación Sanitaria Hospital 12 de Octubre (i+12), Madrid, Spain.

[3] Universidad Complutense de Madrid, Faculty of Medicine, Department of Pediatrics, Madrid, Spain.

Amsterdam University Medical Center (Amsterdam UMC), University of Amsterdam

Principal Investigator: T.W. (Taco) Kuijpers MD PhD [1,2] (all activities)

Co-investigators: M. (Martijn) van de Kuip MD PhD [1] (infectious disease), A.M. (Marceline) van Furth MD PhD [1] (infectious disease), J.M. (Merlijn) van den Berg MD PhD [1] (inflammatory disease)

Hospital Team (all activities): Giske Biesbroek MD PhD [1], Floris Verkuil MD (PhD student) [1], Carlijn (C.W.) van der Zee MD (start 1/8/2022, PhD student) [1]

Recruitment: Dasja Pajkrt MD PhD [1], Michael Boele van Hensbroek MD PhD [1], Dieneke Schonenberg MD [1], Mariken Gruppen MD [1], Sietse Nagelkerke MD PhD [1,2], medical students

Laboratory Team: Machiel H Jansen [1], Ines Goetschalckx (PhD student) [2]

Author Affiliations:

[1] Amsterdam UMC, Emma Children's Hospital, Dept of Pediatric Immunology, Rheumatology and Infectious Disease, University of Amsterdam, The Netherlands

[2] Sanquin, Dept of Molecular Hematology, University Medical Center, Amsterdam, The Netherlands

Bambino Gesù Children’s Hospital (Rome-Italy)

Principal Investigator: Lorenza Romani[1], Maia De Luca[1]

Recruitment Team: Sara Chiurchiù[1], Costanza Tripiciano[1], Stefania Mercadante[1]

Affiliation: 1 Infectious Disease Unit, Academic Department of Pediatrics, Bambino Gesù Children's Hospital, IRCCS, Rome 00165, Italy

ERASMUS MC-Sophia Children's Hospital

Principal Investigator: Clementien L. Vermont[2]

Research group: Henriëtte A. Moll[1], Dorine M. Borensztajn[1], Nienke N. Hagedoorn, Chantal Tan [1], Joany Zachariasse [1], Medical students [1]

Additional investigator: W Dik [3]

[1] Erasmus MC-Sophia Children's Hospital, Department of General Paediatrics, Rotterdam, the Netherlands

[2] Erasmus MC-Sophia Children's Hospital, Department of Paediatric Infectious Diseases & Immunology, Rotterdam, the Netherlands

[3] Erasmus MC, Department of immunology, Rotterdam, the Netherlands

TAIWAN

Principal Investigator: Ching-Fen, Shen, Department of Pediatrics, National Cheng Kung University Hospital, College of Medicine, National Cheng Kung University, Tainan, Taiwan

Riga Stradins University (Riga, Latvia)

Principal Investigator: Dace Zavadska [1,2] (all activities)

Co-investigators: Sniedze Laivacuma [1,3] (adult cohorts)

Recruitment team: Aleksandra Rudzate [1,2], Diana Stoldere [1,2], Arta Barzdina [1,2], Elza Barzdina [1,2], Sniedze Laivacuma[1,3], Monta Madelane [1,3]

Laboratory: Dagne Gravele [2], Dace Svile[2]

Author Affiliations:

[1] Riga Stradins University, Riga, Latvia

[2] Children clinical university hospital, Riga, Latvia

[3] Riga East clinical university hospital, Riga, Latvia

Assistance Publique - Hôpitaux de Paris

Principal Investigator: Romain Basmaci [1,2]

Co-investigator: Noémie Lachaume [1]

Recruitment team: Pauline Bories [1], Raja Ben Tkhayat [1], Laura Chériaux [1], Juraté Davoust [1], Kim-Thanh Ong [1], Marie Cotillon [1], Thibault de Groc [1], Sébastien Le [1], Nathalie Vergnault [1], Hélène Sée [1], Laure Cohen [1], Alice de Tugny [1], Nevena Danekova [1]

Author Affiliations:

[1] Service de Pédiatrie-Urgences, AP-HP, Hôpital Louis-Mourier, F-92700 Colombes, France

[2] Université Paris Cité, Inserm, IAME, F-75018 Paris, France

BioMérieux

Principal Investigator: Marine Mommert-Tripon

Co-investigator: Karen Brengel-Pesce

Author Affiliations: bioMérieux - Open Innovation & Partnerships Department, Lyon, France

University Medical Centre Ljubljana, Slovenia

Principal Investigator: Marko Pokorn [1,2,3]

Co-Investigator: Mojca Kolnik[2]

Research Group (in alphabetical order): Tadej Avčin[2,3], Tanja Avramoska[2], Natalija Bahovec[1], Petra Bogovič[1], Lidija Kitanovski[2,3], Mirijam Nahtigal[1], Lea Papst[1], Tina Plankar Srovin[1], Franc Strle[1,2], Katarina Vincek[1].

Affiliations:

1. Department of Infectious diseases, University Medical Centre Ljubljana, Slovenia
2. University Children's Hospital, University Medical Centre Ljubljana, Slovenia
3. Faculty of Medicine, University of Ljubljana, Slovenia
4. Centre for Clinical research, University Medical Centre Ljubljana

University Medical Center Utrecht, Utrecht, The Netherlands

Principal Investigator: Michiel van der Flier[1,5] (Pediatric Infectious Diseases and Immunology)

Co-investigators: Wim J.E. Tissing[5] (Pediatric Oncology), Roelie M. Wösten-van Asperen[2] (Pediatric Intensive Care Unit), Sebastiaan J Vastert[3] (Pediatric Rheumatology), Daniel C Vijlbrief[4] (Pediatric Neonatal Intensive Care), Louis J. Bont[1,5] (Pediatric Infectious Diseases and Immunology)

PhD student: Coco R. Beudeker[1,5] (Pediatric Infectious Diseases and Immunology)

Affiliations:

1.Pediatric Infectious Diseases and Immunology, 2. Pediatric Intensive Care Unit 3. Pediatric Rheumatology 4. Pediatric Neonatal Intensive Care, Wilhelmina Children's Hospital, University Medical Center Utrecht, Utrecht, The Netherlands

5. Princess Maxima Center for Pediatric Oncology, Utrecht, The Netherlands

University of Bern Partner, Inselspital, Bern University Hospital, University of Bern, Switzerland

Principal Investigator: Philipp Agyeman[1]

Co-Investigators: Christoph Aebi[1], Nina Schöbi[1]

Recruitment team: Mariama Usman[1], Stefanie Schlüchter[1]

[1] Department of Pediatrics, Inselspital, Bern University Hospital, University of Bern, Switzerland

University of Zürich Partner, Kinderspital Zürich, University Children's Hospital Zurich

Principal Investigator: Luregn Schlapbach[1,2]

Co-Investigators: Cornelia Hagmann[1], Florian Zapf[1], Philipp Baumann[1], Barbara Brotschi[1]

Recruitment team: Elisa Zimmermann[1] PhD, Marion Meier[1], Kathrin Weber[1]

[1] Department of Intensive Care and Neonatology, and Children`s Research Center, University Children`s Hospital Zurich, Zurich, Switzerland

[2] Child Health Research Centre, The University of Queensland, Brisbane, Australia

Micropathology Ltd, The Venture Center, University of Warwick Science Park, Sir William Lyons Road, Coventry, CV4 7EZ

Principle Investigator: Prof Colin Fink

Co Investigators: Marie Voice, Leo Calvo-Bado, Michael Steele, Jennifer Holden, Andrew Taylor, Ronan Calvez

Research group: Catherine Davies, Benjamin Evans, Jake Stevens, Peter Matthews, Kyle Billing

Medical University of Graz, Austria (MUG)

Principal Investigator: Werner Zenz[1] (all activities)

Co-investigators (in alphabetical order): Alexander Binder[1] (grant application), Benno Kohlmaier[1] (study design, recruitment), Daniela S. Kohlfürst[1] (study design), Nina A. Schweintzger[1] (all activities), Christoph Zurl[1] (study design, recruitment)

Recruitment team, data managers, laboratory work (in alphabetical order): Susanne Hösele[1], Piyush G. Gampawar[1], Barbara Kapo[1], Manuel Leitner[1], Lena Pölz[1], Alexandra Rusu[1], Glorija Rajic[1], Bianca Stoiser[1], Martina Strempfl[1], Manfred G. Sagmeister[1]

Clinical recruitment partners (in alphabetical order): Sebastian Bauchinger[1], Martin Benesch[3], Astrid Ceolotto[1], Ernst Eber[2], Siegfried Gallistl[1], Harald Haidl[1], Almuthe Hauer[1], Christa Hude[1], Andreas Kapper[7], Markus Keldorfer[5], Sabine Löffler[5], Tobias Niedrist[6], Heidemarie Pilch[5], Andreas Pfleger[2], Klaus Pfurtscheller[4], Siegfried Rödl[4], Andrea Skrabl-Baumgartner[1], Volker Strenger[3], Elmar Wallner[7]

Author Affiliations:

[1] Department of Pediatrics and Adolescent Medicine, Division of General Pediatrics, Medical University of Graz, Graz, Austria

[2]Department of Pediatric Pulmonology, Medical University of Graz, Graz, Austria

[3]Department of Pediatric Hematooncology, Medical University of Graz, Graz, Austria

[4]Paediatric Intensive Care Unit, Medical University of Graz, Graz, Austria

[5]University Clinic of Pediatrics and Adolescent Medicine Graz, Medical University Graz, Graz, Austria

[6]Clinical Institute of Medical and Chemical Laboratory Diagnostics, Medical University Graz, Graz, Austria

[7]Department of Internal Medicine, State Hospital Graz II, Location West, Graz, Austria

Project partner BBMRI-ERIC

Maike K. Tauchert, Biobanking and BioMolecular Resources Research Infrastructure - European Research Infrastructure Consortium (BBMRI-ERIC), Neue Stiftingtalstrasse 2/B/6, 8010, Graz, Austria

LMU Munich Partner (Germany)

Principal Investigator: Ulrich von Both[1,2] MD, FRCPCH (all activities)

Research group: Laura Kolberg[1] MSc (all activities), Patricia Schmied[1] (Study physician), Ioanna Mavridi[1] (PhD student), Irene Alba-Alejandre[3] MD (Study physician)

Clinical recruitment partners (in alphabetical order): Katharina Danhauser, MD[6], Nikolaus Haas, MD[11], Florian Hoffmann, MD[10], Matthias Griese, MD[7], Tobias Feuchtinger, MD[5], Sabrina Juranek, MD[4], Matthias Kappler, MD[7], Eberhard Lurz, MD[8], Esther Maier, MD[4], Karl Reiter, MD[10], Carola Schoen, MD[10], Sebastian Schroepf, MD[9]

Author Affiliations:

[1] Division of Pediatric Infectious Diseases, Department of Pediatrics, Dr. von Hauner Children's Hospital, University Hospital, LMU Munich, Munich, Germany

[2] German Center for Infection Research (DZIF), Partner Site Munich, Munich, Germany

[3] Department of Gynecology and Obstetrics, University Hospital, LMU Munich, Munich, Germany

[4] Division of General Pediatrics, Department of Pediatrics, Dr. von Hauner Children's Hospital, University Hospital, LMU Munich, Munich, Germany

[5] Division of Pediatric Haematology & Oncology, Department of Pediatrics, Dr. von Hauner Children's Hospital, University Hospital, LMU Munich, Munich, Germany

[6] Division of Pediatric Rheumatology, Department of Pediatrics, Dr. von Hauner Children's Hospital, University Hospital, LMU Munich, Munich, Germany

[7] Division of Pediatric Pulmonology, Department of Pediatrics, Dr. von Hauner Children's Hospital, University Hospital, LMU Munich, Munich, Germany

[8] Division of Pediatric Gastroenterology, Department of Pediatrics, Dr. von Hauner Children's Hospital, University Hospital, LMU Munich, Munich, Germany

[9] Neonatal Intensive Care Unit, Department of Pediatrics, Dr. von Hauner Children's Hospital, University Hospital, LMU Munich, Munich, Germany

[10] Paediatric Intensive Care Unit, Department of Pediatrics, Dr. von Hauner Children's Hospital, University Hospital, LMU Munich, Munich, Germany

[11] Department of Pediatric Cardiology and Pediatric Intensive Care, University Hospital, LMU Munich, Germany

London School of Hygiene and Tropical Medicine (LSHTM)

Principal Investigator: Shunmay Yeung [1,2,3]

Research group: Manuel Dewez[1] David Bath[3], Elizabeth Fitchett[1], Fiona Cresswell[1]

1. Clinical Research Department, Faculty of Infectious and Tropical Disease, London School of Hygiene and Tropical Medicine, London
2. Department of Paediatrics, St. Mary's Imperial College Hospital, London
3. Department of Global Health and Development, Faculty of Public Health and Policy, London School of Hygiene and Tropical Medicine, London

Medical Research Council Unit The Gambia at LSHTM, Fajara, The Gambia

Site Principal Investigator: Effua Usuf

Additional Investigators: Kalifa Bojang (Co Investigator), Anna Roca (Co Investigator), Isatou Sarr (Senior Scientist), Momodou Saidykhan (Nurse Co-ordinator), Ebrahim Ndure (Data Manager)

Affiliations: Medical Research Council at LSHTM, Fajara

European Bioinformatics Institute (EMBL-EBI), United Kingdom

Co-investigators: Pedro Madrigal (Bioinformatics) [1], Silvie Fexova (Data curation) [1]

Affiliation: 1 EMBL-EBI, Wellcome Genome Campus, Hinxton, Cambridgeshire, CB10 1SD, UK

Department of Pediatric Infectious Diseases, Medical University of Bialystok, Poland

Site Principal Investigator: Artur Sulik[1]

Co-Investigators: Kacper Toczylowski[1], Dawid Lewandowski[1]

[1]Department of Pediatric Infectious Diseases, Medical University of Bialystok, Poland

SUPPLEMENTAL DIGITAL CONTENT 2. Sample Collection

A research blood sample was taken as soon as feasible after presentation to Patan Hospital. Research samples were obtained as soon as possible after presentation to hospital. When potential participants were identified, parents or guardians were approached by trained research staff in the emergency department or in the pediatric ward. Parents/guardians were given verbal and written information about the study, and they were given time to ask questions, read the information and decide if they wanted their child to be included in the study. Written assent was obtained from children 12-14 years of age.

In situations where the patient required emergency management or it was unsafe for the research staff to approach the participant, a deferred consent approach was used. This approach allowed for research samples to be taken before informed consent was obtained. As soon as possible after research samples were taken using the deferred consent approach, parents/guardians were approached and written informed was sought. If informed consent was given, the participants were included in the study and the study procedures were continued. If informed consent was refused, study samples which had been obtained were destroyed and the participant was not included in any further study procedures.

Blood samples were collected in a syringe. The first 1 mL of blood was transferred directly to an RNA stabilization tube (RNA samples used for other analyses, not relevant to the results reported in this article). The next 2 mLs of blood were transferred to the EDTA tube. The next 1 mL of blood was transferred to the serum tube. Any remaining blood was divided between EDTA and serum tubes. Blood collected in the ethylenediaminetetraacetic acid (EDTA) tube was used for molecular testing. A nasopharyngeal swab sample was obtained and placed in a cryovial with transport medium containing skim milk, tryptone, glucose and glycerol (STGG). Research samples were taken at the same time as clinical samples whenever possible.

Sample processing

When the EDTA sample arrived in the laboratory, an aliquot of whole blood was taken from the sample. The remaining EDTA sample was centrifuged, plasma samples were aliquoted, and a cell pellet sample was stored. All aliquots were frozen at -80°C.

When the nasopharyngeal swab sample arrived in the laboratory an aliquot of STGG was taken and cultured for pneumococcal serotypes, using the Quellung method. This was part of another study running at the same site. A second aliquot of STGG was obtained and stored at -80°C.

Molecular testing

An aliquot of STGG from each nasopharyngeal sample was sent to Micropathology Ltd., University of Warwick Science Park, UK for molecular testing. A whole blood sample from the EDTA tube was also sent to Micropathology Ltd., UK for molecular testing.

An aliquot of plasma was tested using the Siemens Tropical Fever Core panel at Patan Hospital. If a plasma sample was positive for dengue on PCR, this sample was tested using the Siemens Dengue Differentiation panel.

Total nucleic acid was extracted from the nasopharyngeal swab STGG media, and from the blood samples. Nucleic acid from the nasopharyngeal samples was analyzed using the NxTAG™ Respiratory Pathogen Panel + SARS-CoV-2 (Luminex® Corporation). This panel detects 17 different viral targets and 3 bacterial targets.

Extracted nucleic acid from blood samples was analyzed using a molecular diagnostic panel developed and validated at Micropathology Ltd. This panel allows the detection of 9 different viruses (9 viral targets), 16 bacteria (23 targets), and 2 fungal species (6 fungal targets). See Supplement Digital Content 3 for the full list of pathogen targets.

Molecular testing at Patan Hospital

Extraction of nucleic acid

After the plasma sample was thawed, the sample was combined with lysis buffer and isopropanol. The mixture was transferred to an absorption column and centrifuged. An internal control was then added. The first wash buffer was added, and the solution was centrifuged. The liquid was then discarded, and the second wash buffer was added to the absorption tube and then centrifuged again. The liquid was discarded again; the absorption column was allowed to dry before RNase-free water was added. Following a final centrifuge step, the extracted nucleic acid solution was either used immediately or stored at -80°C.

Tropical Fever Core Panel

The Siemens Tropical Fever Core Panel is designed to identify DNA sequences associated with specific pathogens, using plasma or serum samples. This panel is recommended for research-use only currently. Targeted RNA is reverse transcribed to complementary DNA (cDNA). Using real-time PCR, the DNA molecules are then simultaneously amplified. If increased fluorescence is detected from the probe, this indicates the presence of the specific DNA sequences of interest.

The Siemens Tropical Fever Core Panel has seven pathogen targets: dengue virus, West Nile virus, chikungunya, *Rickettsia species*, *Salmonella species*, *Plasmodium species* and *Leptospira species*.

The PCR master-mix was prepared by combining the primer-probe, enzyme and buffer supplied in the kit. Then, 10 µl of master-mix was added to the wells on the PCR plate. Next, 10 µl of extracted nucleic acid, positive control, or negative control was added to each well. The plate was loaded into the PCR machine (BioRad CFX96) and the PCR program was run. Results were reviewed using the BioRad software.

Dengue Serotype Panel

If a sample was positive for dengue on the Tropical Fever Core Panel, extracted nucleic acid from that sample was analyzed using the Siemens Dengue Differentiation Panel. This

panel uses the same principle as the Tropical Fever Panel, except the targets are the four dengue serotypes. The steps undertaken to run the Dengue Serotype Panel are like the Tropical Fever Core Panel steps described above.

SUPPLEMENTAL DIGITAL CONTENT 3. Additional molecular panels used in the study.

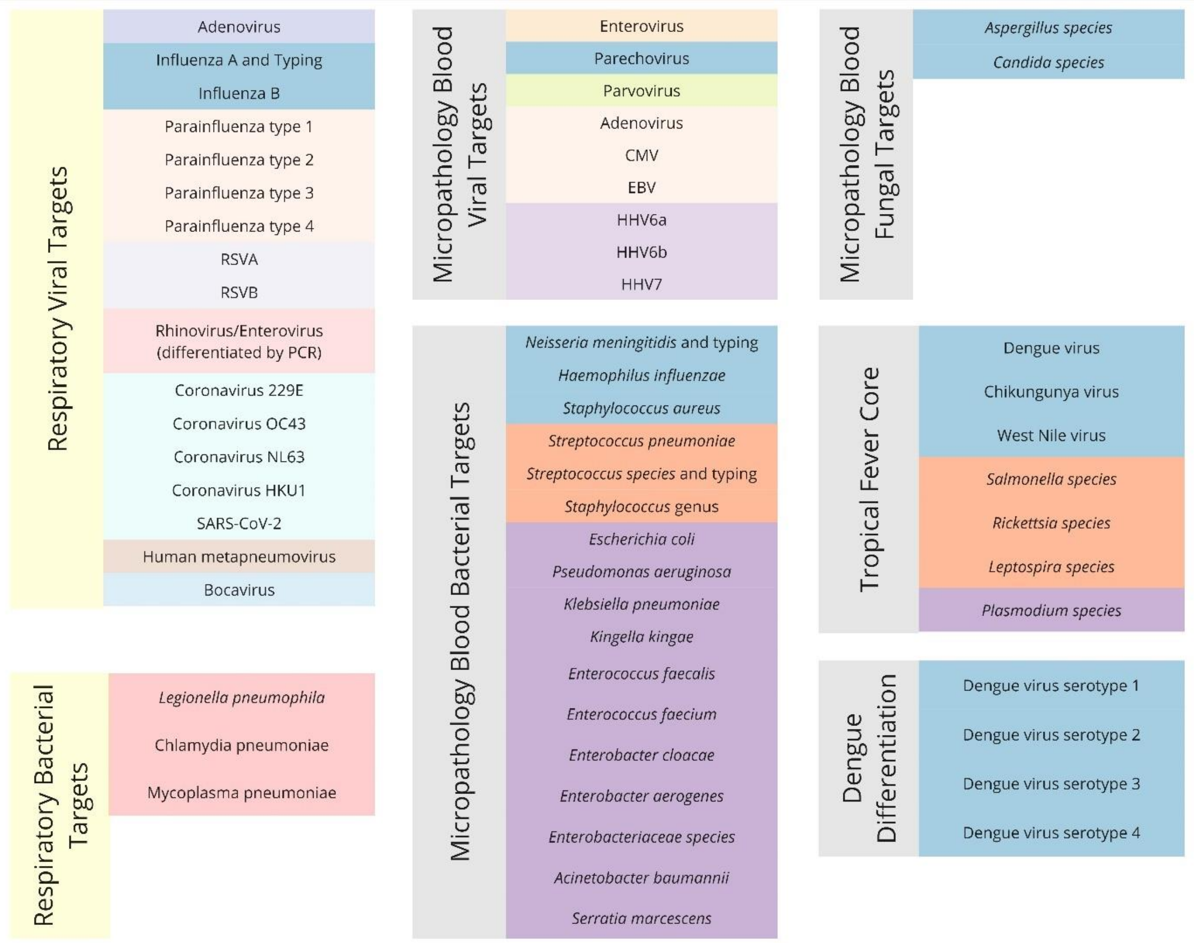

Nasopharyngeal samples were tested using the NxTag Respiratory Panel + SARS-CoV-2. Whole blood samples were tested using the in-house blood molecular panel at the laboratory of Micropathology Ltd. Plasma samples were tested using the Siemens Tropical Fever Core Panel and the Dengue Differentiation Panel at Patan Hospital. RSV, respiratory syncytial virus; SARS-CoV-2, severe acute respiratory syndrome coronavirus 2; CMV, cytomegalovirus; EBV, Epstein–Barr virus; HHV, human herpesvirus.

SUPPLEMENTAL DIGITAL CONTENT 4. Additional molecular testing to diagnose the causes of infection

Samples underwent molecular testing for different targets. Only a subset of these molecular targets has been associated with clinical disease in case-control studies; these targets were used to identify additional causes of infection. The rationale for which targets used is explained in the following paragraphs.

Shah et al.[1] reported results from molecular testing in a large cohort of European children admitted to hospital with febrile illnesses. The Shah et al. study used the same molecular targets as two of the molecular panels used in our study (NxTAG™ Respiratory Pathogen Panel and the Micropathology Ltd. Blood Panel); they compared molecular results between bacterial and viral cases. *Streptococcus pneumoniae*, *Neisseria meningitidis*, Group A *Streptococcus* and *Escherichia coli* had significantly higher detection rates in the bacterial group compared with the viral group. Positive molecular results for these four bacteria were found to be associated with bacterial infections in other published reports as well.[2-9] Bacterial targets from the Tropical Fever Core panel were also used to re-classify cases, based on studies using PCR testing in rickettsial disease, leptospirosis, and salmonellosis.[10-12]

The viral targets used as possibly clinically significant were dengue virus and enterovirus in blood, and respiratory syncytial virus (RSV) (types A and B), influenza (types A and B), parainfluenza (types 1, 3 and 4) and human metapneumovirus in nasopharyngeal samples. These viral targets were chosen based on the findings of large case-control trials.[1, 13, 14]

Use of additional molecular testing in identifying possible causes of infection

Following a positive result for one of the pathogen targets identified above, the pathogen was assigned as the likely cause of infection if the following criteria were met. Results from nasopharyngeal and blood samples were treated differently.

For positive molecular nasopharyngeal results, cases were only re-classified if:

1. Case had a respiratory presentation (cough, increased work of breathing, increased respiratory rate, supplementary oxygen, diagnosis of respiratory tract infection)
2. Case not originally classified as "definite bacterial" or "probable bacterial"

For positive additional molecular blood results, cases were re-classified if:

1. Molecular result can explain all or part of the clinical syndrome
2. Another investigation does not explain all the clinical syndrome

Statistical methods

Data were analyzed using R version 4.3.1. For normally distributed data, mean and standard deviation are reported. Median and inter-quartile range are presented for non-normally distributed data. A sample size estimation of 600-800 participants was chosen to allow identification of a broad range of different infectious causes.

REFERENCES

1. Shah P, Voice M, Calvo-Bado L, et al. Relationship between molecular pathogen detection and clinical disease in febrile children across Europe: a multicentre, prospective observational study. *Lancet Reg Health Eur* 2023; 32: 100682. 20230726. DOI: 10.1016/j.lanepe.2023.100682.
2. Rello J, Lisboa T, Lujan M, et al. Severity of pneumococcal pneumonia associated with genomic bacterial load. *Chest* 2009; 136: 832-840. 20090511. DOI: 10.1378/chest.09-0258.
3. Iroh Tam PY, Hernandez-Alvarado N, Schleiss MR, et al. Detection of Streptococcus pneumoniae from culture-negative dried blood spots by real-time PCR in Nigerian children with acute febrile illness. *BMC Res Notes* 2018; 11: 657. 20180910. DOI: 10.1186/s13104-018-3770-2.
4. Troendle M and Pettigrew A. A systematic review of cases of meningitis in the absence of cerebrospinal fluid pleocytosis on lumbar puncture. *BMC Infectious Diseases* 2019; 19: 692. DOI: 10.1186/s12879-019-4204-z.
5. Pardo J, Klinker KP, Borgert SJ, et al. Detection of Neisseria meningitidis from negative blood cultures and cerebrospinal fluid with the FilmArray blood culture identification panel. *J Clin Microbiol* 2014; 52: 2262-2264. 20140416. DOI: 10.1128/jcm.00352-14.
6. Guiducci S, Moriondo M, Nieddu F, et al. Culture and Real-time Polymerase Chain reaction sensitivity in the diagnosis of invasive meningococcal disease: Does culture miss less severe cases? *PLOS ONE* 2019; 14: e0212922. DOI: 10.1371/journal.pone.0212922.

7. Obiero CW, Gumbi W, Mwakio S, et al. Detection of pathogens associated with early-onset neonatal sepsis in cord blood at birth using quantitative PCR. *Wellcome Open Res* 2022; 7: 3. 20221108. DOI: 10.12688/wellcomeopenres.17386.3.
8. Lucignano B, Ranno S, Liesenfeld O, et al. Multiplex PCR allows rapid and accurate diagnosis of bloodstream infections in newborns and children with suspected sepsis. *J Clin Microbiol* 2011; 49: 2252-2258. 20110406. DOI: 10.1128/jcm.02460-10.
9. Tsalik EL, Jones D, Nicholson B, et al. Multiplex PCR to diagnose bloodstream infections in patients admitted from the emergency department with sepsis. *J Clin Microbiol* 2010; 48: 26-33. 20091021. DOI: 10.1128/jcm.01447-09.
10. Watthanaworawit W, Turner P, Turner C, et al. A prospective evaluation of real-time PCR assays for the detection of Orientia tsutsugamushi and Rickettsia spp. for early diagnosis of rickettsial infections during the acute phase of undifferentiated febrile illness. *Am J Trop Med Hyg* 2013; 89: 308-310. 20130603. DOI: 10.4269/ajtmh.12-0600.
11. de Abreu Fonseca C, Teixeira de Freitas VL, Caló Romero E, et al. Polymerase chain reaction in comparison with serological tests for early diagnosis of human leptospirosis. *Trop Med Int Health* 2006; 11: 1699-1707. DOI: 10.1111/j.1365-3156.2006.01727.x.
12. Neupane DP, Dulal HP and Song J. Enteric Fever Diagnosis: Current Challenges and Future Directions. *Pathogens* 2021; 10 20210401. DOI: 10.3390/pathogens10040410.
13. O'Brien KL, Baggett HC, Brooks WA, et al. Causes of severe pneumonia requiring hospital admission in children without HIV infection from Africa and Asia: the PERCH multi-country case-control study. *The Lancet* 2019; 394: 757-779. DOI: 10.1016/S0140-6736(19)30721-4.
14. Rhedin S, Lindstrand A, Hjelmgren A, et al. Respiratory viruses associated with community-acquired pneumonia in children: matched case-control study. *Thorax* 2015; 70: 847-853. 20150615. DOI: 10.1136/thoraxjnl-2015-206933.

SUPPLEMENTAL DIGITAL CONTENT 5. List of pathogens contributing to confirmed causes of infection.

Several cases had more than one pathogen identified. *Source of the positive test was either the respiratory PCR panel (NxTAG), the blood PCR panel (Micropathology Ltd.), the Tropical Core panel (Siemens) or routinely available rests. PCR, polymerase chain reaction; IgM, immunoglobulin M, IgG, immunoglobulin G.

**Pathogen**	**Total number of cases**	**Source of positive test***
**Viral pathogens**	181	
Dengue	101	Blood antigen/PCR
RSV	30	Respiratory PCR
Influenza	25	Respiratory PCR
SARS-CoV-2	17	Respiratory PCR
Human metapneumovirus	14	Respiratory PCR
Parainfluenza	8	Respiratory PCR
Enterovirus	7	Blood PCR
Hepatitis A	4	IgM/IgG hepatitis A
**Bacterial pathogens**	42	
E. coli	20	Urine culture/blood culture
S. pneumoniae	5	Blood culture/blood PCR
S. Paratyphi A	4	Blood culture
S. Typhi	3	Blood culture/CSF culture
Klebsiella species	3	Urine culture
M. tuberculosis	2	Gene Xpert
O. tsutsugamushi	2	Scrub typhus IgM
N. meningiditis	2	Blood PCR
S. aureus	1	Pleural fluid culture
